# Deciphering the Dilemma: Choosing the Optimal Total Neoadjuvant Treatment Strategy for Locally Advanced Rectal Cancer

**DOI:** 10.3390/curroncol31080320

**Published:** 2024-07-29

**Authors:** Erik Manriquez, Sebastián Solé, Javiera Silva, Juan Pablo Hermosilla, Rubén Romero, Felipe Quezada-Diaz

**Affiliations:** 1 Complejo Asistencial Doctor Sotero del Rio, Santiago 8207257, Chile; ejmanriquez@uc.cl; 2 Clínica IRAM, Santiago 7630370, Chile; sebastian.sole@iram.cl (S.S.); javiera.silva@iram.cl (J.S.); 3 Radiotherapy Oncology Program, School of Medicine, Universidad Diego Portales, Santiago 8370191, Chile; drjphermosilla@gmail.com (J.P.H.); ruben.romero@hospitaldipreca.cl (R.R.); 4 Hospital Dipreca, Santiago 7601003, Chile; 5 Clínica Universidad de los Andes, Santiago 7620157, Chile; 6 Center for Cancer Prevention and Control (CECAN), Santiago 8380453, Chile

**Keywords:** neoadjuvant therapy, rectal cancer, total neoadjuvant therapy, chemoradiation, short course radiotherapy

## Abstract

Rectal cancer management has evolved significantly, particularly with neoadjuvant treatment strategies. This narrative review examines the development and effectiveness of these therapies for locally advanced rectal cancer (LARC), highlighting the historical quest that led to current neoadjuvant alternatives. Initially, trials showed the benefits of adding radiotherapy (RT) and chemotherapy (CT) to surgery, reducing local recurrence (LR). The addition of oxaliplatin to chemoradiotherapy (CRT) further improved outcomes. TNT integrates chemotherapy and radiotherapy preoperatively to enhance adherence, timing, and systemic control. Key trials, including PRODIGE 23, CAO/ARO/AIO 12, OPRA, RAPIDO, and STELLAR, are analyzed to compare short-course and long-course RT with systemic chemotherapy. The heterogeneity and difficulty in comparing TNT trials due to different designs and outcomes are acknowledged, along with their promising long-term results. On the other hand, it briefly discusses the potential for non-operative management (NOM) in select patients, a strategy gaining traction due to favorable outcomes in specific trials. As a conclusion, this review underscores the complexity of rectal cancer treatment, emphasizing individualized approaches considering patient preferences and healthcare resources. It also highlights the importance of interpreting impressive positive or negative results with caution due to the variability in study designs and patient populations.

## 1. Introduction

Colorectal cancer (CRC) is the third most commonly diagnosed cancer, and the second-leading cause of cancer deaths worldwide [1]. Its treatment places a heavy economic burden on healthcare providers, patients, and families [2]. Rectal cancer, in particular, is the 10th most deadly, with 310,000 deaths, constituting 3.2% of all cancer deaths [3]. The diagnosis, treatment, and survival of rectal cancer present global challenges [4,5].

The current treatment of rectal cancer involves a multidisciplinary approach [6], achieving improved local and promising better distant outcomes. From the initial studies incorporating radiotherapy (RT) and chemotherapy (CT), both in combination and separately, many questions have been answered, while new ones have emerged.

The role of each component of this multimodal approach and the different methods of delivering these treatments remain subjects of debate [6]. Additionally, global social and economic challenges demand cost-effective interventions to achieve optimal cancer care, especially in low- to middle-income countries, ensuring optimal value for money in healthcare planning to address health inequalities worldwide [7].

The objective of this review is to comprehensively explore the evolution of neoadjuvant therapy for rectal cancer by examining the currently available alternatives, considering patient heterogeneity and the differences among trials regarding inclusion criteria and delivered treatments. The goal is to promote better decision-making in a patient-centered approach, taking into consideration patients’ preferences as well as healthcare providers’ resource availability.

## 2. A Long Ride

**Figure 1:**
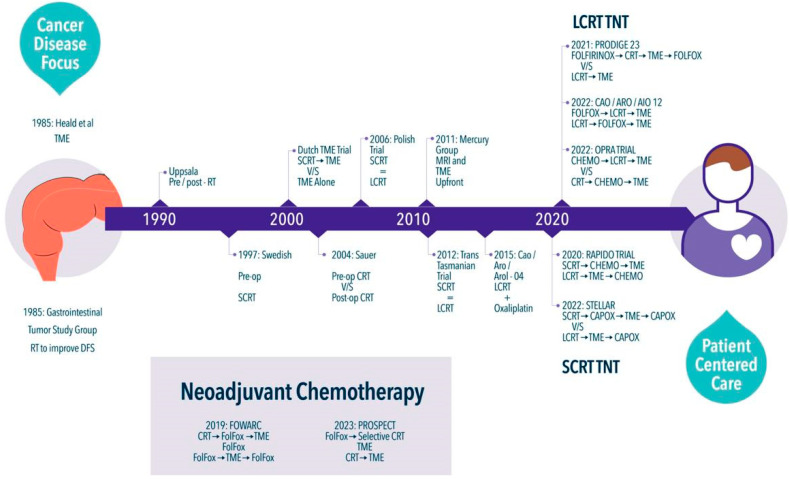
Evolution of rectal cancer treatment [8,9,11,12,13,14,15,16,17,18,19,20,21,22,23] CRT: Chemoradiotherapy, CT or Chemo: Chemotherapy, DFS: Disease-free Survival LCRT: Long Course Chemoradiotherapy, SCRT: Short Course Radiotherapy, TME: Total Mesorectal Excision, TNT: Total Neoadjuvant Therapy.

The current debate regarding rectal cancer treatment has emerged with the advent of new multimodal alternatives. However, the history of rectal cancer treatment spans several decades (Figure 1). One of the first randomized trials conducted decades ago, showed promising results in terms of cancer recurrence when adding RT and CT as adjuvants after surgery [8]. The study demonstrated a recurrence rate of 55% for the surgery-alone group compared to 33% for the RT combined with the CT group. In parallel, Heald et al. published their first results of the standardized total mesorectal excision (TME) technique in 1986 [9], with a five-year local recurrence (LR) rate of 3.7%. Over the next decade, this technique showed robust oncologic results, improving five-year overall survival (OS) from 38% to 68% [10].

During the next decade, changes in the timing of RT showed improved LR rates with the implementation of neoadjuvant short-course radiotherapy (SCRT) as demonstrated by Uppsala et al. [11], the Swedish Rectal Cancer Trial [12], and the Dutch TME trial [13]. The latter, published in 2001, reported an LR rate at two years of 2.4% in the SCRT plus TME group vs. 8.2% in the TME only group (*p* < 0.001).

In addition to the discoveries of the last 20 years, the quest to optimize multimodality treatment led to the comparison of different neoadjuvant regimens. The administration of long-course chemoradiation therapy (LCRT) to achieve local control showed good results but no OS benefits, as demonstrated by Sauer et al., who compared preoperative chemoradiotherapy (CRT) by randomly assigning patients with clinical stage T3 or T4 or node-positive disease to receive either preoperative or postoperative CRT. The overall five-year survival rates were 76% and 74%, respectively (*p* = 0.80). The five-year cumulative incidence of local relapse was 6% for patients assigned to preoperative CRT and 13% in the postoperative-treatment group (*p* = 0.006) [14]. Similar results were found by the EORTC Radiotherapy Group Trial 22921 in 2006, where the addition of either preoperative or postoperative 5-fluorouracil to 45 Gy administered in 5 weeks conferred better local control, improving LR from 17.1% in the no-CT group vs. 7.6–9.6% in pre- or post- or pre + postoperative CT during a five-year follow-up. However, there was no improvement in OS or systemic recurrence [24]. 

These findings led to the comparison of both strategies. Two of the most important trials in this regard were the Polish Colorectal Study Group randomized controlled trial (RCT) published in 2006, which randomized 312 patients to receive either preoperative SCRT (25 Gy in five fractions of 5 Gy) and surgery within 7 days or chemoradiation (50.4 Gy in 28 fractions of 1.8 Gy, bolus 5-fluorouracil, and leucovorin) and surgery 4 to 6 weeks later. The study showed that neoadjuvant chemoradiation did not increase survival, local control, or late toxicity compared with SCRT alone [15]. On the other hand, the Trans-Tasmanian Trial, published in 2012, enrolled patients with cT3N0-2M0 rectal adenocarcinoma. SCRT consisted of pelvic RT 5 × 5 Gy in 1 week, early surgery, and six courses of adjuvant CT. LCRT was 50.4 Gy, 1.8 Gy/fraction, in 5.5 weeks, with continuous infusional fluorouracil 225 mg/m^2^ per day, surgery in 4 to 6 weeks, and four courses of CT. The study showed that three-year LR rates between SCRT and LCRT were not statistically significantly different, nor were other oncologic outcomes or toxicity [16].

In this global scenario, questions arose about the role of each component of multimodality treatment and the timing of their delivery. However, the evolution of understanding rectal cancer behavior and advances in diagnostic tools brought new elements to the discussion. One of the most relevant advances occurred at the beginning of the last decade when the MERCURY group, in 2011, identified different patient groups based on their magnetic resonance features. This allowed for the appropriate selection of patients who could potentially “omit” neoadjuvant therapy and proceed directly to TME surgery [17]. They showed that a “good” prognosis included MRI-predicted safe circumferential resection margins, with MRI-predicted T2/T3a/T3b (less than 5 mm spread from the muscularis propria), regardless of MRI N stage, could be operated directly, having an LR rate of 3% at five years of follow-up. This selective approach was reproduced by different groups worldwide, as shown by the OCUM Rectal Cancer Trial [25] and the QuickSilver Clinical Trial [26] with favorable results.

In conclusion, over more than 20 years, several strategies in the neoadjuvant and adjuvant approach, patient selection, and staging methods evolved, dramatically improving local control, with rates of LR below 10%. However, OS did not show significant changes, remaining consistent with rates achieved after TME implementation in the late 1990s [10].

## 3. Time to Make a Change

Up until the past decade, the role of adjuvant chemotherapy in treating rectal cancer remained uncertain [27]. Several phase III studies, such as the interim analysis of PETACC6 [28], NSABP R-04 [29], and STAR 01 [30], explored the benefit of adding oxaliplatin to LCRT in order to optimize treatment with no benefits in terms of OS. However, the German Study CAO/ARO/AIO-04 showed an increase in the pathological complete response (pCR) rate in patients receiving neoadjuvant LCRT with additional oxaliplatin (17% vs. 13%). This effect was also associated with better three-year disease-free survival (DFS) (75.9% vs. 71.2%) [18]. Later, the ADORE trial [31] showed that LCRT followed by TME and adjuvant FOLFOX improved DFS in patients with rectal cancer ypStage II and III disease after preoperative LCRT (68% vs. 57% at six years of follow-up). Even though the introduction of oxaliplatin, as a neoadjuvant or adjuvant treatment, showed no benefits and even more toxicity in some trials (22–24), the potential benefits shown in the latest mentioned trials propelled the tendency to adopt the use of LCRT with additional oxaliplatin as part of neoadjuvant or adjuvant treatment as the standard of care in patients with locally advanced rectal cancer (LARC), particularly in the American Guidelines (National Comprehensive Cancer Network (NCCN) 2018) [32]. However, European guidelines tailor LARC treatment depending on risk factors, allowing up-front surgery for cT3 in the absence of risk factors and restricting the use of LCRT or SCRT, with or without FOLFOX preoperatively, to more advanced LARC cases [33]

Given this background, the need emerged to explore alternative methods of delivering oxaliplatin-based CT to enhance adherence, improve timing, and achieve better systemic control and outcomes. Hence, the concept of total neoadjuvant therapy (TNT) emerged.

## 4. Total Neoadjuvant Therapy

In recent years, in selected cases, particularly in Europe, it has been accepted that some LARC patients, which include cT3–T4 and/or cN1–N2, can be operated on upfront, avoiding neoadjuvant toxicity and achieving good oncologic outcomes [33]. However, there are LARC patients whose local and systemic control must be optimized by a multimodality approach. The TNT concept implies delivering RT and systemic CT preoperatively. Recently published trials aimed to identify the appropriate neoadjuvant treatment strategies in conjunction with the most suitable patients for each modality and sequence of treatment, which may include SCRT, LCRT, and CT, alone or combined, administered at different preoperative times. 

Several trials conducted over the last decade explored this topic, recruiting patients during similar time periods. While they had similarities regarding the main outcomes, the enrolled patients were not similar in terms of tumor location and the presence of risk factors. Additionally, the treatment strategies differed, mainly due to variations in CT doses and cycles, as well as RT delivery strategies.

## 5. LCRT-Based TNT

One of the most relevant datasets is from the PRODIGE 23 trial, whose three-year follow-up results were published in 2021. The trial randomized 231 patients to the experimental arm (FOLFIRINOX for six cycles, followed by CRT, TME, and adjuvant CT with FOLFOX for three cycles). The standard-of-care group included 230 patients who received CRT followed by TME and adjuvant CT for six months. Patients included in this trial had confirmed rectal adenocarcinoma within 15 cm of the anal verge, stage cT3 (at risk of LR and for which a multidisciplinary tumor board recommended preoperative CRT) or cT4. In the experimental arm, almost 60% had mid or high-rectum cancer, 81% were T3 stage, 90% had N(+) status, and only 21% had a radial margin less than or equal to 1 mm. Extramural vascular invasion (EMVI) was not reported. The control arm had similar features. The primary endpoint was three-year DFS, which was favorable in the experimental arm (76% [95% CI 69 to 81] vs. 69% [62 to 74]). There was a significant difference in metastasis-free survival (MFS) (79% vs. 72%), but no differences in OS (91% in the experimental arm vs. 88% in the control group) or locoregional relapse (4% in the experimental arm vs. 6% in the control group). pCR was significantly better in the experimental group (28% vs. 12%). There were no differences in serious adverse events during the entire treatment period [34]. More recently, presented but not published, updated data from seven years of follow-up showed significant improvements in all oncologic outcomes, including MFS (65% vs. 73%) and OS (76.1% vs. 81.9%) for the TNT group [19].

In 2022, the CAO/ARO/AIO 12 group published their long-term results. Patients included had rectal adenocarcinoma up to 12 cm above the anal verge; cT3 tumor less than 6 cm from the anal verge, cT3 cancer in the middle third of the rectum (≥6–12 cm) with extramural tumor spread into the mesorectum of more than 5 mm (>cT3b), cT4 tumors, or lymph node involvement. They were randomized to group A for induction CT before CRT or to group B for consolidation CT after CRT, followed by TME in both groups. Induction and consolidation CT consisted of FOLFOX for three cycles. CRT included 5-fluorouracil and oxaliplatin. In the consolidation arm, 78% were T3 stage, 90% had N(+), 22% had a circumferential resection margin (CRM) (+), and nearly 90% had mid or low cancers. EMVI status was not reported. The main outcome was pCR, which was achieved in 17% of group A vs. 25% of group B, indicating that only the consolidation group met the significant criteria [20]. In a secondary analysis, the three-year DFS was 73% in both groups (hazard ratio, 0.95; 95% CI, 0.63–1.45, *p* = 0.82); the three-year cumulative incidence of locoregional recurrence (6% vs. 5%, *p* = 0.67) and distant metastases (18% vs. 16%, *p* = 0,52) were not significantly different. Chronic toxicity grades 3 to 4 occurred in 10 of 85 patients (11.8%) in group A and 8 of 66 patients (9.9%) in group B at three years. The authors concluded that CRT followed by CT resulted in higher pCR without compromising DFS, toxicity, quality of life, or stool incontinence and is thus proposed as the preferred TNT sequence if organ preservation is a priority [35].

Lastly, the OPRA trial published its first results in 2022 [21], enrolling patients with clinical stage II (T3–4, N0) or stage III (any T, N1–2) who were treated with induction CT followed by CRT or CRT followed by consolidation CT and either TME or watch-and-wait based on tumor response. Patients in both groups received four months of FOLFOX or CAPOX and 5000 to 5600 cGy of radiation combined with either the continuous infusion of fluorouracil or capecitabine during RT. The enrolled patients’ features were as follows: cT3 stage between 76–78%, N(+) 70–72%, and a median tumor distance from the anal verge of 4.3–4.5 cm. CRM and EMVI were not reported. The primary outcome was DFS compared with a historical cohort. At three years, DFS was 76% (95% CI, 69 to 84) for the induction group and 76% (95% CI, 69 to 83) for the consolidation group, in line with the three-year DFS rate (75%) observed historically [21]. At five years, updated data showed the estimated five-year DFS rates were 71% (95% CI, 64 to 79) for the induction group and 69% (95% CI, 62 to 77) for the consolidation group. The estimated five-year OS rates were also similar in both groups; 88% (95% CI, 83 to 94) for the induction group and 85% (95% CI, 79 to 91) for the consolidation group. Estimated five-year local recurrence-free survival (LRFS) rates were (94% [95% CI, 90 to 98] for the induction group and 90% [95% CI, 85 to 96] for the consolidation group) and distant metastasis-free survival (DMFS) rates were similar in both groups (80% [95% CI, 74 to 87] for the induction group and 78% [95% CI, 71 to 85] for the consolidation group) [36]. However, the most interesting and promising secondary outcome of this trial was organ preservation, as it was designed to offer a watch-and-wait strategy to patients who achieved a complete clinical response (cCR) after receiving TNT. The proportion of patients who achieved organ preservation at five years in the intention-to-treat population was 39% (95% CI, 32 to 48) for the induction group and 54% (95% CI, 46 to 62) for the consolidation group (*p* = 0.012). The percentage of patients who received sphincter-preserving surgery was similar between those who had TME after restaging (39/71 [55%]) and those who had TME after tumor regrowth (28/64 [44%]; *p* = 0.23). Five-year DFS was also similar for patients who underwent TME after restaging (64% [95% CI, 53 to 78]) and those who underwent TME after tumor regrowth. These impressive findings must be interpreted cautiously because they are not the main outcome, and the trial was not designed to explore them specifically. Furthermore, they could be explained by the use of TNT, the overall treatment length, and the assessment of tumor response almost eight weeks from the end of TNT, even offering organ preservation to patients with a near-complete response but being aware of the possibility of regrowth [36].

## 6. SCRT-Based TNT

On the other hand, the STELLAR trial, a non-inferiority trial published in 2022, randomized a TNT group (298 patients) to receive short-term RT (5 Gy × 5 days) followed by four cycles of CAPOX at 7–14 days after completion of RT. The CRT (293 patients) group received 50 Gy in 25 fractions over five weeks, concurrently with capecitabine. Postoperative CT comprised two cycles of CAPOX in the TNT group or six cycles of CAPOX in the CRT group. TME was recommended in both groups 6–8 weeks after preoperative treatment. The included patients had cT3-T4 and/or N(+) status, located in the distal or middle third of the rectum. In the TNT group, 82% of patients were T3 stage, 86% had N(+) status, nearly all patient’s tumors were located 10 cm or less from the anal verge, 56% had mesorectal fascia (MRF)(+), and 53% had EMVI(+). The trial confirmed non-inferiority for the primary endpoint, three-year DFS, in patients receiving TNT vs. standard CRT (64.5% vs. 62.3%, respectively; *p* < 0.001 for non-inferiority). OS was better in the TNT group (86% vs. 75%, *p* < 0.05). There were no differences in locoregional recurrence (LRR) (8.4% in the TNT group vs. 11% in the CRT group) or distant metastasis (22% in the TNT group vs. 24% in the CRT group). The total rate of pCR and sustained cCR in the TNT group was 21.8%, significantly higher than in the CRT group (12.3%, *p* = 0.002). The compliance rate was higher in the TNT group but was associated with an approximately two-fold rate of grade 3-plus toxicity compared with CRT (26.5% vs. 12.6%) [22].

Similarly, the RAPIDO Trial, published in 2020 with three-year follow-up results, enrolled patients with primary, locally advanced rectal adenocarcinoma, classified as high risk on pelvic MRI (with at least one of the following criteria: clinical tumor [cT] stage cT4a or cT4b, EMVI, clinical nodal [cN] stage cN2, involved MRF, or enlarged lateral lymph nodes). Eligible participants were randomly assigned (1:1) to either the experimental or standard-of-care group. Those in the experimental treatment group received SCRT (5 × 5 Gy over a maximum of 8 days), followed by six cycles of CAPOX CT or nine cycles of FOLFOX, and then TME. Patients in the standard-of-care group received 28 daily fractions of 1.8 Gy up to 50.4 Gy or 25 fractions of 2.0 Gy up to 50.0 Gy, with concomitant twice-daily oral capecitabine, followed by TME. If stipulated by hospital policy, they also received adjuvant CT with eight cycles of CAPOX or 12 cycles of FOLFOX4. The primary endpoint was three-year disease-related treatment failure (DrTF), defined as the first occurrence of locoregional failure, distant metastasis, new primary colorectal tumor, or treatment-related death. Unlike previously described trials, this trial included higher-risk patients, with the TNT group having 66% of patients with two or more risk features of those described in the inclusion criteria. Sixty-one percent of rectal tumors were located 10 cm or below from the anal verge. Moreover, nearly 60% of patients in each arm received adjuvant CT at the treating center’s discretion. At five years, the cumulative probability of DrTF was 27.8% (95% CI, 23.7 to 31.8) in the TNT group and 34.0% (95% CI, 29.6 to 38.4) in the CRT group (HR: 0.79 [95% CI, 0.63 to 1.00]; *p* = 0.0480). The cumulative probability of distant metastasis in the TNT group was 23.0% and 30.4% in the CRT group (HR: 0.73 [95% CI, 0.57 to 0.93]; *p* = 0.011). The cumulative probability of OS was 81.7% in the TNT group compared with 80.2% in the CRT group (HR: 0.91 [95% CI, 0.70 to 1.19]; *p* = 0.50). A higher rate of LRR was detected in the TNT group compared with the CRT group; 44/431 (10.2%) and 26/428 (6.1%), respectively, *p* = 0.027 [23]. With regard to pCR, this occurred in 28% of 423 patients in the TNT group compared with 14% of 398 in the CRT group (OR 2.37 [95% CI 1.67 to 3.37, *p* < 0.0001]) [37].

This last trial is the most controversial due to its higher LRR in the TNT group, which raised concerns from different parts of the world [38,39]. The possible explanations for this event, according to the authors, could be attributed to heterogeneous RT techniques used in different centers, a higher proportion of breached mesorectum in the TNT group (possibly due to different timing of surgery in each group), surgical planning changes post-restaging translated into more sphincter-sparing surgery, which could leave microscopic carcinoma in situ and tumor fragmentation. Additionally, the omission of poor responding patients who could have experienced tumor growth during TNT treatment, as restaging assessment was carried out at the end of neoadjuvant treatment, allowing the progression of aggressive cancers [23].

It is worth noting that the patients included in RAPIDO correspond to a higher-risk group compared to the rest of the trials published in the TNT era. Moreover, RAPIDO showed a significant difference regarding DrTF, explained mainly by distant metastasis control. Furthermore, it achieved a pCR of 28%, which was double that of the control group. Considering this, it is important to highlight that LRR in this trial represented a post hoc analysis, which should be interpreted cautiously as it could be caused by one of the reasons previously mentioned or even by chance [40]. Hence, we assert that this does not warrant a robust enough rationale to discard this strategy, especially when contemplating the trials discussed previously, including those such as the Polish II trial [41].

As more evidence is accumulated, it is crucial to consider the conclusions from the RAPIDO authors if deciding to use TNT based on SCRT. This means incorporating an early response assessment with interruption of the weakest part of the treatment, i.e., the CT, in case no response or even progression is seen. Additionally, adequate coverage of the tumor cell-containing tissue volumes, dose escalation, increased rate of lateral lymph node dissection on the indication, and a surgical plan based on the initial pretreatment MRI should be considered [23].

When considering these two SCRT trials, it is important to note that from a cost-effectiveness point of view, SCRT could be a more efficient strategy regarding resource usage. However, if the intention is to avoid an abdominoperineal resection, LCRT schemes would be the most cost-effective option [42].

## 7. Neoadjuvant Chemotherapy

While the previously mentioned trials tried to define the best sequence and timing to deliver and combine RT and CT, some groups tried to diminish RT toxicity by delivering just CT as TNT. The PROSPECT trial, which was published in 2023, aimed to answer this issue. This was a non-inferiority, randomized trial of neoadjuvant FOLFOX (with CRT given only if the primary tumor decreased in size by <20% or if FOLFOX was discontinued due to side effects) compared with CRT. Included adults with rectal cancer who had been cT2 node-positive, T3 node-negative, or T3 node-positive, who were candidates for sphincter-sparing surgery. The primary endpoint was DFS. Half of the patients had a non-palpable tumor, and approximately 85% of all patients had a middle or high cancer, without T4 stage. Neither CRM nor EMVI were reported. At a median follow-up of 58 months, FOLFOX was non-inferior to CRT in terms of DFS (hazard ratio for disease recurrence or death, 0.92; 90.2% CI, 0.74 to 1.14; *p* = 0.005 for non-inferiority). Five-year DFS was 80.8% (95% CI, 77.9 to 83.7) in the FOLFOX group and 78.6% (95% CI, 75.4 to 81.8) in the CRT group. pCR was similar in both groups (21.9% in the FOLFOX group and 24.3% in the CRT group). About 10% of FOLFOX patients received CRT either pre- or postoperatively [43]. One main issue regarding this trial was related to the included patients, as many of them were described as having a good prognosis defined by the Mercury Group [17], so could be treated with upfront TME according to some guidelines [33].

On the other hand, the FOWARC trial [44], whose final results were published in 2019, enrolled stage II and III cancer patients, about 25% of whom were cT4, with a majority being N(+) and 30% with CRM(+). Similarly, this trial compared an exclusive neoadjuvant strategy with a FOLFOX scheme followed by surgery and adjuvant FOLFOX, against LCRT, followed by TME and infusional fluorouracil. The third arm consisted of FOLFOX and LCRT followed by TME and adjuvant FOLFOX. The probability of three-year DFS was 73.5%, 72.9%, and 77.2%, respectively (*p* = 0.709). The three-year probability of LR after R0/1 resection was 8.3%, 8.0%, and 7.0%, respectively (*p* = 0.873). The three-year OS rate was 90.7%, 91.3%, and 89.1%, respectively (*p* = 0.971). The study concluded that there was no difference regarding any of these alternatives. Table 1 summarizes the main findings of the described trials.

## 8. Non-Operative Management (NOM)

The evolution of treatment, the consistent rate of pCR shown in different trials, even higher with TNT modalities, alongside the unexpected cCR occurring in some patients have raised considerable interest in understanding and applying organ preservation as a valid alternative. Its discussion is beyond the scope of this review but it is worth mentioning that it has been added as part of treatment algorithms in the latest guidelines (NCCN 2024), but only in dedicated centers. Moreover, our understanding and ability to choose and follow up with those patients is critical. We are looking expectantly at the evolution of the described trials. Considering that there are some reported experiences on NOM with SCRT [45,46] as well, upcoming trials should help us improve our patient selection for this approach [47,48].

## 9. Microsatellite Instability-High (MSI-H) / Deficient Mismatch Repair (dMMR) in LARC Patients

During the last decade, CT was the only systemic treatment available for advanced CRC. With growing research on deficient mismatch repair (dMMR) and microsatellite instability-high (MSI-H), evidence of CT response in these patients has been inconsistent, showing heterogeneous outcomes [49]. Consequently, immunotherapy has been explored to improve oncologic outcomes in these patients [50].

The most relevant trials are Keynote 177 and Checkmate 142. The Keynote 177 trial randomized 307 patients with metastatic MSI-H/dMMR CRC who had not previously received treatment to receive either pembrolizumab or CT (5-fluorouracil-based therapy with or without bevacizumab or cetuximab). After a median follow-up of 32.4 months, pembrolizumab was superior to CT in terms of progression-free survival (median, 16.5 vs. 8.2 months; hazard ratio, 0.60; 95% confidence interval [CI], 0.45 to 0.80; *p* = 0.0002) [51]. The Checkmate 142 trial enrolled patients with no prior treatment in the metastatic setting for MSI-H/dMMR CRC, treating them with nivolumab every two weeks plus low-dose ipilimumab every six weeks until disease progression. The primary endpoint was the objective response rate, which was 69% (95% CI, 53 to 82) with a 13% complete response rate [52].

Approximately 5 to 10% of rectal adenocarcinomas are mismatch-repair deficient, and these tumors respond poorly to standard CT regimens, including neoadjuvant CT in locally advanced rectal cancer [53,54]. A recent phase 2 trial aimed to investigate the overall response and frequency of sustained clinical complete response to neoadjuvant treatment with dostarlimab, a PD-1 inhibitor. Twelve patients completed treatment with dostarlimab and had at least six months of follow-up, all showing a clinical complete response (100%; 95% confidence interval, 74 to 100) [55].

Acknowledging the current evidence, the NCCN 2024 guidelines prefer checkpoint inhibitor immunotherapy for up to six months (dostarlimab-gxly, nivolumab, or pembrolizumab) in MSI-H/dMMR LARC over CT. Therefore, it is recommended to promptly identify the MSI status of every LARC patient to deliver a more personalized treatment [56].

## 10. Which Neoadjuvant Treatment Is the Best?

Rectal cancer is a complex disease, and there is no one-size-fits-all treatment strategy. To date, TNT is the preferred strategy for LARC. However, there has been no trial comparing different TNT strategies head-to-head, particularly between SCRT and LCRT TNT. Moreover, the patients included are not fairly comparable to the existing studies. Despite the RAPIDO Trial including patients with the worst features, the primary endpoint was achieved, with distant control and OS quite similar to other trials. Concerns were raised regarding LRR for SCRT TNT but this should be interpreted cautiously since it corresponds to a post hoc analysis. Moreover, the LRR using SCRT was addressed by the STELLAR trial, which showed better 3-year OS without compromising local control. Thus, it seems there is room for SCRT TNT schemes, particularly if NOM is not the main objective, in limited-resource environments, and when prompt initiation of systemic treatment is desirable. This should be conducted while considering the recommendations made by the RAPIDO authors.

On the other hand, LCRT TNT schemes used in trials are heterogeneous, with quite similar oncologic outcomes. The most impressive results are related to the organ preservation rate exhibited by the OPRA trial, which significantly increased the chance of providing NOM for some patients. However, there are no other trials showing the same results; thus, it is important to have more evidence before adopting this approach as a routine practice.

Finally, it is noteworthy that rectal cancer patients are heterogeneous, and current classification and staging systems do not allow us to appropriately identify the best candidates for any of the described modalities. The differences among trials could be explained by the presence of higher or lower proportions of high-risk patients, the different RT techniques used to deliver SCRT, the amount of CT received, and the main outcomes for which each trial was designed. It is important to consider patients’ preferences, beliefs, and values alongside the availability of resources at each center. Understanding the individual and social aspects involved in a shared decision-making process is crucial. This often means considering pragmatic, logistic, and economic factors in our daily practice.

## Figures and Tables

**Table 1:** Trials summary.

Trial	Regimen	Main outcome		Secondary	Outcomes	
		3y DFS *	3y MFS *	3y OS	pCR *	3y LRR
PRODIGE 23	CT → LCRT → TME → CT	76%	79%	91%	28%	4%
N = 461	LCRT → TME → CT	69%	72%	88%	12%	6%
		pCR	3y DFS	3y OS	3y DM	3y LRR
CAO/ARO/AIO 12	CT → LCRT → TME	17%	73%	92%	18%	6%
N = 306	LCRT → CT → TME	25% *	73%	92%	16%	5%
		5y DFS	5y MFS	5y OS	5y OP *	5y LRFS
OPRA	CT → LCRT → TME or WW	71%	80%	88%	39%	94%
N = 324	LCRT → CT → TME or WW	69%	78%	85%	54%	90%
		5y DrTF *	5y DM *	5y OS	pCR *	5y LRR *
RAPIDO	SCRT → CT → TME	27.8%	23%	81.7%	28%	10.2%
N = 920	LCRT → TME → CT	34%	30.4%	80.2%	14%	6.1%
		3y DFS	3y DM	3y OS *	pCR+cCR *	3y LRR
STELLAR	SCRT → CT → TME → CT	64.5% (non inferior)	22%	86%	21.8%	8.4%
N = 591	LCRT → TME → CT	62.3%	24%	75%	12.3%	11%
		5y DFS		5y OS	pCR	5y LRR
PROSPECT	CT → Selective LCRT → TME ± CT	80.8% (non inferior)	-	89.5%	21.9%	1.8%
N = 1128	LCRT → TME ± CT	78.6%	-	90.2%	24.3%	1.6%
		3y DFS		3y OS	pCR	3y LRR
FOWARC	LCRT → TME → 5FU	72.9%	-	91.3%	14%	8%
N = 495	LCRT + CT → TME → CT	77.2%	-	89.1%	27.5%	7%
	CT → TME → CT	73.5%	-	90.7%	6.5%	8.3%

* Significant difference. cCR: Complete clinical response, CT: Chemotherapy, DFS: Disease-free Survival, DM: Distant metastasis, DrTF: Disease-related treatment failure, LCRT: Long Course Chemoradiotherapy, LRR;Locoregional recurrence, MFS: Metastasis-free Survival, OP: Organ preservation, OS: Overall survival. pCR: Pathological complete response, SCRT: Short Course Radiotherapy, TME: Total Mesorectal Excision, TNT: Total Neoadjuvant Therapy, WW: Watch and wait.

## Abbreviation/Glossary List

cCR	Complete Clinical Response
CRC	Colorectal Cancer
CRM	Circumferential Resection Margin
CRT	Chemoradiotherapy
CT	Chemotherapy
DFS	Disease-free Survival
EMVI	Extramural Vascular Invasion
LARC	Locally Advanced Rectal Cancer
LCRT	Long Course Chemoradiotherapy
LR	Local Recurrence
MFS	Metastasis-free Survival
MRF	Mesorectal Fascia
NAT	Neoadjuvant Therapy
OS	Overall Survival
pCR	Pathological Complete Response
RCT	Randomized Controlled Trial
RT	Radiotherapy
SCRT	Short Course Radiotherapy
TME	Total Mesorectal Excision
TNT	Total Neoadjuvant Therapy

## References

[1] Morgan E., Arnold M., Gini A., Lorenzoni V., Cabasag C.J., Laversanne M., Vignat J., Ferlay J., Murphy N., Bray F. (2023). Global burden of colorectal cancer in 2020 and 2040: Incidence and mortality estimates from GLOBOCAN. Gut.

[2] Bhimani N., Wong G.Y., Molloy C., Dieng M., Hugh T.J. (2022). Cost of colorectal cancer by treatment type from different health economic perspectives: A systematic review. Eur. J. Surg. Oncol..

[3] Rawla P., Sunkara T., Barsouk A. (2019). Epidemiology of colorectal cancer: Incidence, mortality, survival, and risk factors. Prz. Gastroenterol..

[4] Galandiuk S. (2023). Rectal Cancer: New Challenges. Dis. Colon Rectum.

[5] McMullen C.K., Bulkley J.E., Altschuler A., Wendel C.S.M., Grant M.R., Hornbrook M.C., Sun V.R., Krouse R.S. (2016). Greatest Challenges of Rectal Cancer Survivors: Results of a Population-Based Survey. Dis. Colon Rectum.

[6] Smith H.G., Nilsson P.J., Shogan B.D., Harji D., Gambacorta M.A., Romano A., Brandl A., Qvortrup C. (2024). Neoadjuvant treatment of colorectal cancer: Comprehensive review. BJS Open..

[7] Khan S.Z., Lengyel C.G. (2023). Challenges in the management of colorectal cancer in low- and middle-income countries. Cancer Treat. Res. Commun..

[8] Gastrointestinal Tumor Study Group (1985). Prolongation of the disease-free interval in surgically treated rectal carcinoma. N. Engl. J. Med..

[9] Heald R.J., Ryall R.D. (1986). Recurrence and survival after total mesorectal excision for rectal cancer. Lancet.

[10] Heald R.J., Moran B.J., Ryall R.D., Sexton R., MacFarlane J.K. (1998). Rectal cancer: The Basingstoke experience of total mesorectal excision, 1978–1997. Arch Surg..

[11] Påhlman L., Glimelius B. (1990). Pre- or postoperative radiotherapy in rectal and rectosigmoid carcinoma. Report from a randomized multicenter trial. Ann. Surg..

[12] Trial S.R.C., Cedermark B., Dahlberg M., Glimelius B., Påhlman L., E Rutqvist L., Wilking N. (1997). Improved survival with preoperative radiotherapy in resectable rectal cancer. N. Engl. J. Med..

[13] Kapiteijn E., Marijnen C.A., Nagtegaal I.D., Putter H., Steup W.H., Wiggers T., Rutten H.J., Pahlman L., Glimelius B., Van Krieken J.H. (2001). Preoperative radiotherapy combined with total mesorectal excision for resectable rectal cancer. N. Engl. J. Med..

[14] Sauer R., Becker H., Hohenberger W., Rödel C., Wittekind C., Fietkau R., Martus P., Tschmelitsch J., Hager E., Hess C.F. (2004). Preoperative versus postoperative chemoradiotherapy for rectal cancer. N. Engl. J. Med..

[15] Bujko K., Nowacki M.P., Nasierowska-Guttmejer A., Michalski W., Bebenek M., Kryj M. (2006). Long-term results of a randomized trial comparing preoperative short-course radiotherapy with preoperative conventionally fractionated chemoradiation for rectal cancer. Br. J. Surg..

[16] Ngan S.Y., Burmeister B., Fisher R.J., Solomon M., Goldstein D., Joseph D., Ackland S.P., Schache D., McClure B., McLachlan S.-A. (2012). Randomized trial of short-course radiotherapy versus long-course chemoradiation comparing rates of local recurrence in patients with T3 rectal cancer: Trans-Tasman Radiation Oncology Group trial 01.04. J. Clin. Oncol..

[17] Taylor F.G.M., Quirke P., Heald R.J., Moran B., Blomqvist L., Swift I., Sebag-Montefiore D.J., Tekkis P., Brown G. (2011). Preoperative high-resolution magnetic resonance imaging can identify good prognosis stage I, II, and III rectal cancer best managed by surgery alone: A prospective, multicenter, European study. Ann. Surg..

[18] Rödel C., Graeven U., Fietkau R., Hohenberger W., Hothorn T., Arnold D., Hofheinz R.-D., Ghadimi M., Wolff H.A., Lang-Welzenbach M. (2015). Oxaliplatin added to fluorouracil-based preoperative chemoradiotherapy and postoperative chemotherapy of locally advanced rectal cancer (the German CAO/ARO/AIO-04 study): Final results of the multicentre, open-label, randomised, phase 3 trial. Lancet Oncol..

[19] Conroy T., Etienne P.L., Rio E., Evesque L., Mesgouez-Nebout N., Vendrely V., Artignan X., Bouche O., Boileve A., Delaye M. (2023). Total neoadjuvant therapy with mFOLFIRINOX versus preoperative chemoradiation in patients with locally advanced rectal cancer: 7-year results of PRODIGE 23 phase III trial, a UNICANCER GI trial. J. Clin. Oncol..

[20] Fokas E., Allgäuer M., Polat B., Klautke G., Grabenbauer G.G., Fietkau R., Kuhnt T., Staib L., Brunner T., Grosu A.-L. (2019). Randomized Phase II Trial of Chemoradiotherapy Plus Induction or Consolidation Chemotherapy as Total Neoadjuvant Therapy for Locally Advanced Rectal Cancer: CAO/ARO/AIO-12. J. Clin. Oncol..

[21] Garcia-Aguilar J., Patil S., Gollub M.J., Kim J.K., Yuval J.B., Thompson H.M., Verheij F.S., Omer D.M., Lee M., Dunne R.F. (2022). Organ Preservation in Patients with Rectal Adenocarcinoma Treated with Total Neoadjuvant Therapy. J. Clin. Oncol..

[22] Jin J., Tang Y., Hu C., Jiang L.M., Jiang J., Li N., Liu W.-Y., Chen S.-L., Li S., Lu N.-N. (2022). Multicenter, Randomized, Phase III Trial of Short-Term Radiotherapy Plus Chemotherapy versus Long-Term Chemoradiotherapy in Locally Advanced Rectal Cancer (STELLAR). J. Clin. Oncol..

[23] Dijkstra E.A., Nilsson P.J., Hospers G.A.P., Bahadoer R.R., Meershoek-Klein Kranenbarg E., Roodvoets A.G.H., Putter H., Berglund A., Cervantes A., Crolla R.M.P.H. (2023). Locoregional Failure During and after Short-course Radiotherapy followed by Chemotherapy and Surgery Compared with Long-course Chemoradiotherapy and Surgery: A 5-Year Follow-up of the RAPIDO Trial. Ann. Surg..

[24] Bosset J.F., Collette L., Calais G., Mineur L., Maingon P., Radosevic-Jelic L., Daban A., Bardet E., Beny A., Ollier J.-C. (2006). Chemotherapy with preoperative radiotherapy in rectal cancer. N. Engl. J. Med..

[25] Ruppert R., Junginger T., Ptok H., Strassburg J., Maurer C.A., Brosi P., Sauer J., Baral J., Kreis M., Wollschlaeger D. (2018). Oncological outcome after MRI-based selection for neoadjuvant chemoradiotherapy in the OCUM Rectal Cancer Trial. Br. J. Surg..

[26] Kennedy E.D., Simunovic M., Jhaveri K., Kirsch R., Brierley J., Drolet S., Brown C., Vos P.M., Xiong W., MacLean T. (2019). Safety and Feasibility of Using Magnetic Resonance Imaging Criteria to Identify Patients with “Good Prognosis” Rectal Cancer Eligible for Primary Surgery: The Phase 2 Nonrandomized QuickSilver Clinical Trial. JAMA Oncol..

[27] Breugom A.J., Swets M., Bosset J.F., Collette L., Sainato A., Cionini L., Glynne-Jones R., Counsell N., Bastiaannet E., Broek C.B.M.v.D. (2015). Adjuvant chemotherapy after preoperative (chemo)radiotherapy and surgery for patients with rectal cancer: A systematic review and meta-analysis of individual patient data. Lancet Oncol..

[28] Schmoll H.J., Haustermans K., Price T.J., Nordlinger B., Hofheinz R., Daisne J., Janssens J., Brenner B., Schmidt P., Reinel H. (2014). Preoperative Chemoradiotherapy and Postoperative Chemotherapy with Capecitabine +/− Oxaliplatin in Locally Advanced Rectal Cancer: Interim Analysis for Disease-Free Survival of Petacc 6. Ann. Oncol..

[29] O’Connell M.J., Colangelo L.H., Beart R.W., Petrelli N.J., Allegra C.J., Sharif S., Pitot H.C., Shields A.F., Landry J.C., Ryan D.P. (2014). Capecitabine and oxaliplatin in the preoperative multimodality treatment of rectal cancer: Surgical end points from National Surgical Adjuvant Breast and Bowel Project trial R-04. J. Clin. Oncol..

[30] Aschele C., Lonardi S., Cionini L., Pinto C., Cordio S.S., Rosati G., Bianchi A.S., Tagliagambe A., Frisinghelli M., Zagonel V. (2016). Final results of STAR-01: A randomized phase III trial comparing preoperative chemoradiation with or without oxaliplatin in locally advanced rectal cancer. J. Clin. Oncol..

[31] Hong Y.S., Kim S.Y., Lee J.S., Nam B.H., Kim K.P., Kim J.E., Park Y.S., Park J.O., Baek J.Y., Lee K.-W. (2019). Oxaliplatin-Based Adjuvant Chemotherapy for Rectal Cancer after Preoperative Chemoradiotherapy (ADORE): Long-Term Results of a Randomized Controlled Trial. J. Clin. Oncol..

[32] Benson A.B., Venook A.P., Al-Hawary M.M., Cederquist L., Chen Y.J., Ciombor K.K., Cohen S., Cooper H.S., Deming D., Engstrom P.F. (2018). Rectal Cancer, Version 2.2018, NCCN Clinical Practice Guidelines in Oncology. J. Natl. Compr. Canc Netw..

[33] Glynne-Jones R., Wyrwicz L., Tiret E., Brown G., Rödel C., Cervantes A., Arnold D., ESMO Guidelines Committee (2017). Rectal cancer: ESMO Clinical Practice Guidelines for diagnosis, treatment and follow-up. Ann. Oncol.

[34] Conroy T., Bosset J.F., Etienne P.L., Rio E., François É., Mesgouez-Nebout N., Vendrely V., Artignan X., Bouché O., Gargot D. (2021). Neoadjuvant chemotherapy with FOLFIRINOX and preoperative chemoradiotherapy for patients with locally advanced rectal cancer (UNICANCER-PRODIGE 23): A multicentre, randomised, open-label, phase 3 trial. Lancet Oncol..

[35] Fokas E., Schlenska-Lange A., Polat B., Klautke G., Grabenbauer G.G., Fietkau R., Kuhnt T., Staib L., Brunner T., Grosu A. (2022). Chemoradiotherapy Plus Induction or Consolidation Chemotherapy as Total Neoadjuvant Therapy for Patients with Locally Advanced Rectal Cancer: Long-term Results of the CAO/ARO/AIO-12 Randomized Clinical Trial. JAMA Oncol..

[36] Verheij F.S., Omer D.M., Williams H., Lin S.T., Qin L.X., Buckley J.T., Thompson H.M., Yuval J.B., Kim J.K., Dunne R.F. (2024). Long-Term Results of Organ Preservation in Patients with Rectal Adenocarcinoma Treated with Total Neoadjuvant Therapy: The Randomized Phase II OPRA Trial. J. Clin. Oncol..

[37] Bahadoer R.R., Dijkstra E.A., van Etten B., Marijnen C.A.M., Putter H., Kranenbarg E.M.K., Roodvoets A.G.H., Nagtegaal I.D., Beets-Tan R.G.H., Blomqvist L.K. (2021). Short-course radiotherapy followed by chemotherapy before total mesorectal excision (TME) versus preoperative chemoradiotherapy, TME, and optional adjuvant chemotherapy in locally advanced rectal cancer (RAPIDO): A randomised, open-label, phase 3 trial. Lancet Oncol..

[38] Riou O., Gourgou S., Conroy T. (2023). Comment on “Locoregional Failure During and After Short-Course Radiotherapy Followed by Chemotherapy and Surgery Compared to Long-Course Chemoradiotherapy and Surgery: A Five-Year Follow-Up of the RAPIDO Trial”: The RAPIDO Trial Does Not Achieve Its Primary Endpoint. Ann. Surg. Open.

[39] Vailati B.B., Cerdán-Santacruz C., São Julião G.P., Corbi L., Perez R.O. (2023). Short-Course Radiation and Consolidation Chemotherapy for Rectal Cancer-the Rise and Fall of a Treatment Strategy-Rest in Peace. Dis. Colon Rectum.

[40] Nilsson P.J., van Etten B., Hospers G.A.P., Marijnen C.A.M., Meershoek-Klein Kranenberg E., Roodvoets A.G.H., van de Velde C.J.M., Glimelius B. (2024). Comment on the RAPIDO Trial Point-Counterpoint Debate. Dis. Colon Rectum.

[41] Bujko K., Wyrwicz L., Rutkowski A., Malinowska M., Pietrzak L., Kryński J., Michalski W., Olędzki J., Kuśnierz J., Zając L. (2016). Long-course oxaliplatin-based preoperative chemoradiation versus 5 × 5 Gy and consolidation chemotherapy for cT4 or fixed cT3 rectal cancer: Results of a randomized phase III study. Ann. Oncol..

[42] Raldow A.C., Chen A.B., Russell M., Lee P.P., Hong T.S., Ryan D.P., Cusack J.C., Wo J.Y. (2019). Cost-effectiveness of Short-Course Radiation Therapy vs Long-Course Chemoradiation for Locally Advanced Rectal Cancer. JAMA Netw. Open.

[43] Schrag D., Shi Q., Weiser M.R., Gollub M.J., Saltz L.B., Musher B.L., Goldberg J., Al Baghdadi T., Goodman K.A., McWilliams R.R. (2023). Preoperative Treatment of Locally Advanced Rectal Cancer. N. Engl. J. Med..

[44] Deng Y., Chi P., Lan P., Wang L., Chen W., Cui L., Chen D., Cao J., Wei H., Peng X. (2019). Neoadjuvant Modified FOLFOX6 With or Without Radiation Versus Fluorouracil Plus Radiation for Locally Advanced Rectal Cancer: Final Results of the Chinese FOWARC Trial. J. Clin. Oncol..

[45] Nilsson P.J., Ahlberg M., Kordnejad S., Holm T., Martling A. (2021). Organ preservation following short-course radiotherapy for rectal cancer. BJS Open.

[46] Bujko K., Pietrzak L., Partycki M., Szczepkowski M., Wyrwicz L., Rupiński M., Rutkowski A., Mróz A. (2017). The feasibility of short-course radiotherapy in a watch-and-wait policy for rectal cancer. Acta Oncol..

[47] Verschoor Y.L., Lambregts D.M.J., van den Berg J., Grotenhuis B.A., Aalbers A., Van Triest B., Beets-Tan R.G., van de Belt M., Dokter S., Balduzzi S. (2023). Radiotherapy, atezolizumab, and bevacizumab in rectal cancers with the aim of organ preservation: The TARZAN study. J. Clin. Orthod..

[48] Quezada F.F., Diaz-Feldman L.E., Manriquez E., Caire N., Carvajal G. (2024). No operation after short course radiotherapy followed by consolidation chemotherapy in locally advanced rectal cancer: The prospective, single arm NOAHS-ARC trial. J. Clin. Orthod..

[49] Kavun A., Veselovsky E., Lebedeva A., Belova E., Kuznetsova O., Yakushina V., Grigoreva T., Mileyko V., Fedyanin M., Ivanov M. (2023). Microsatellite Instability: A Review of Molecular Epidemiology and Implications for Immune Checkpoint Inhibitor Therapy. Cancers.

[50] Golshani G., Zhang Y. (2020). Advances in immunotherapy for colorectal cancer: A review. Ther. Adv. Gastroenterol..

[51] André T., Shiu K.-K., Kim T.W., Jensen B.V., Jensen L.H., Punt C., Smith D., Garcia-Carbonero R., Benavides M., Gibbs P. (2020). Pembrolizumab in Microsatellite-Instability-High Advanced Colorectal Cancer. N. Engl. J. Med..

[52] Lenz H.-J., Van Cutsem E., Limon M.L., Wong K.Y.M., Hendlisz A., Aglietta M., García-Alfonso P., Neyns B., Luppi G., Cardin D.B. (2022). First-Line Nivolumab Plus Low-Dose Ipilimumab for Microsatellite Instability-High/Mismatch Repair-Deficient Metastatic Colorectal Cancer: The Phase II CheckMate 142 Study. J. Clin. Oncol..

[53] Cercek A., Fernandes G.D.S., Roxburgh C.S., Ganesh K., Ng S., Sanchez-Vega F., Yaeger R., Segal N.H., Reidy-Lagunes D.L., Varghese A.M. (2020). Mismatch Repair-Deficient Rectal Cancer and Resistance to Neoadjuvant Chemotherapy. Clin. Cancer Res..

[54] Alex A.K., Siqueira S., Coudry R., Santos J., Alves M., Hoff P.M., Riechelmann R.P. (2017). Response to Chemotherapy and Prognosis in Metastatic Colorectal Cancer With DNA Deficient Mismatch Repair. Clin. Color. Cancer.

[55] Cercek A., Lumish M., Sinopoli J., Weiss J., Shia J., Lamendola-Essel M., El Dika I.H., Segal N., Shcherba M., Sugarman R. (2022). PD-1 Blockade in Mismatch Repair-Deficient, Locally Advanced Rectal Cancer. N. Engl. J. Med..

[56] Referenced with permission from the NCCN Clinical Practice Guidelines in Oncology (NCCN Guidelines®) for Rectal Cancer Version 3.2024.© National Comprehensive Cancer Network, Inc. 2024. All rights reserved. Accessed [july 09, 2024]. To view the most recent and complete version of the guideline, go online to NCCN.org.

